# A Dataset of Abdominal CT with Artery and Vein Segmentations for Colorectal Cancer Surgical Planning

**DOI:** 10.1038/s41597-026-07303-2

**Published:** 2026-05-04

**Authors:** Jan Hrubovcak, Petr Strakos, Jan Kubicek, Khyati Sethia, Sumit Kaushik, Milan Jaros, Lubomir Martinek, Marek Penhaker, Jana Bahrova, Marie Chadrabova, Michal Nohel, Lukas Knybel, Tomas Blazek, Jan Migulski, Lubomir Riha

**Affiliations:** 1 Department of Surgery, University Hospital Ostrava, Ostrava, Czech Republic; 2 IT4Innovations, VSB — Technical University of Ostrava, Ostrava, Czech Republic; 3 Department of Cybernetics and Biomedical Engineering, VSB — Technical University of Ostrava, 17. listopadu 2172/15, Ostrava-Poruba, 708 00 Czech Republic; 4 Department of Deputy Director of Science and Research, University Hospital & Faculty of Medicine, Ostrava, 708 52 Czech Republic; 5 Department of Biomedical Engineering, Faculty of Electrical Engineering and Communication, Brno University of Technology, Brno, 616 00 Czech Republic; 6 Department of Oncology, University Hospital & Faculty of Medicine, Ostrava, 708 52 Czech Republic

## Abstract

Safe colorectal cancer (CRC) resection depends on accurate preoperative understanding of mesenteric vascular anatomy, which is highly variable and poorly represented in existing datasets. We curated dual-phase contrast-enhanced abdominal CT (CECT) scans from 60 adults imaged over one year on a Siemens SOMATOM Force scanner (slice thickness 0.75 mm). Arterial phases (25-30 s) and venous phases (55–60 s) were manually segmented in 3D Slicer by an experienced surgeon and verified by a senior colorectal surgeon; without inter-phase registration to preserve native characteristics. The resource includes 60 CECT studies (50 dual-phase, 10 venous only), each containing  ~700–900 axial slices. Deliverables comprise CECT volumes, per-structure 3D label masks for major mesenteric arteries and veins. Example figures of overlays and 3D masks are provided. Intended uses include vessel segmentation, vascular-aware surgical planning, and registration research. Limitations include single-institution acquisition, difficulty delineating small vessels <4 mm, absence of inter-phase registration, and altered vascular anatomy from surgeries.

## Background & Summary

Colorectal cancer (CRC) stands among the most critical oncologic challenges globally, ranking as the third most commonly diagnosed malignancy and the second leading cause of cancer-related mortality. In 2023 alone, it accounted for over 1.9 million new cases and more than 930,000 deaths worldwide, with a continuously increasing burden in low- and middle-income countries. Contrarily, the Czech Republic—historically one of the countries with the highest CRC incidence—has achieved a marked epidemiological shift, largely due to national screening initiatives and the integration of modern diagnostic tools^1–3^.

As of 2023, CRC incidence and mortality rates in the Czech Republic have declined to 57 and 27 per 100,000, respectively—down from 85 and 45 per 100,000 in 2000. This represents a 33% reduction in incidence and a 40% reduction in mortality. The 5-year CRC prevalence exceeds 61,000 individuals, with over 7,000 new cases and 3,300 deaths annually. A cornerstone of this improvement has been the National CRC Screening Program, which now exceeds 50% participation, using modalities such as fecal occult blood testing (FOBT), fecal immunochemical testing (FIT) and screening colonoscopy. The shift toward earlier detection and personalized intervention has significantly improved patient outcomes^4,5^.

Despite these advances, colorectal resection surgery continues to pose a risk of serious complications, including preoperative and postoperative complications due to anatomical variability of the abdominal vasculature. Two particularly vulnerable vascular territories—the splenic flexure (Griffith’s point) and the rectosigmoid junction (Sudeck’s point)—represent classical watershed zones where unrecognized arterial disruption may lead to bowel ischemia or necrosis. Beyond ischemia, intraoperative bleeding due to atypical vascular branching remains a frequent and life-threatening challenge. Moreover, the oncologically correct extent of CRC resection is defined by vascular supply and co-determines prognosis^6^.

Contemporary preoperative imaging, such as contrast-enhanced computed tomography(CECT), enables visualization of the mesenteric vasculature. However, interpreting these scans, particularly the venous-phase imagery, often is outside the expertise of general surgeons. The complex and highly variable nature of colonic vessels, especially in regions such as the hepatic flexure, further complicates surgical planning^7^.

To address this gap, we curated a dataset and developed an artificial intelligence-based system capable of automated three-dimensional segmentation of abdominal arteries and veins using standard dual-phase CECT imaging. The dataset comprises 60 unselected patients scanned on a Siemens SOMATOM Force dual-energy CT system with high-resolution 0.75 mm slice thickness. Arterial and venous phases were captured at 25–30 s and 55–65 s post-contrast injection, respectively. Vascular segmentations—performed by experienced surgeons and validated by senior colorectal specialists—formed the basis for a deep learning model trained to recognize and reconstruct complex anatomical variations.

The term “colorectal cancer surgery” encompasses a heterogeneous group of procedures, and the vascular structures of interest depend on tumor location and the surgical technique used. In right-sided colon cancer, surgeons primarily evaluate branches of the superior mesenteric artery and vein, particularly the ileocolic, right colic, and middle colic vessels, which define the extent of central vascular ligation and lymphadenectomy. In left-sided colon cancer and sigmoid resections, attention shifts to the inferior mesenteric artery and its branches, including the left colic and sigmoid arteries, while rectal surgery requires careful assessment of the superior rectal vessels and pelvic vascular anatomy. The surgical approach may also influence which vessels are considered critical, as techniques such as complete mesocolic excision and central vascular ligation require precise identification of major vascular trunks and their anatomical variants. Although branches supplying the small bowel may be relevant in small-bowel surgery, which is less common than colorectal cancer surgery, these distal branches usually play a limited role in planning standard colorectal resections. Therefore, the annotation strategy in this dataset prioritised vessels most relevant to colorectal oncologic procedures. This supports studies on preoperative planning by accurately mapping patient-specific vascular anatomy, highlighting major arterial and venous structures—such as the superior mesenteric artery and its colic branches, or the splenic and mesenteric venous systems. This tool aims to mitigate the risk of intraoperative hemorrhage and postoperative ischemia. The system’s scope will progressively expand to encompass the full abdominal cavity, ultimately supporting interventions involving the liver, stomach, pancreas, and rectum.

This work also reviews publicly available datasets and studies in Table 1, addressing abdominal vascular imaging, with particular emphasis on their relevance to CRC surgery and artificial intelligence (AI)-based analysis. Accurate depiction of visceral arteries and veins is essential for safe and adequate oncologic resection and preserving adequate tissue perfusion. Current preoperative planning is hindered by pronounced vascular variability and limited surgeon oriented imaging tools. Numerous datasets exist for imaging of CRC or for vascular segmentation in abdominal viscera, but few integrate both domains in a manner applicable to colorectal surgical planning. Here we critically appraise prior efforts—spanning hepatic, mesenteric, and aortic vascular datasets as well as CRC specific radiomics resources—to highlight their scope, strengths, and limitations. This synthesis provides the framework for positioning our work: the introduction of a dual phase CT dataset with comprehensive arterial and venous annotations specifically designed for training and validation of AI algorithms for automated vascular recognition in preoperative CRC resection planning.

**Table 1:** Summary Table of Relevant Studies.

Citation	# Patients	Modality	CRC	Vessel	Vessel Type	Output
Schenk *et al*.^8^	13 pairs	CT+MRI	No	Yes	Portal, hepatic vv.	MRI vs CT seg.
Tong *et al*.^1^	230	CT	Yes	No	—	No vascular analysis, CRC radiomics
Simpson *et al*.^2,18^	197	CT (portal)	Yes	Yes	Hepatic vsl.	Tumor, liver, vessel, FLR seg.
Criscuolo *et al*.^6,10^	30	CT	No	Yes	Abd. vessel Bifurcation & Reg. landmarks	
Yang *et al*.^15^	5	CT	Yes	Yes	Portal, hepatic vv.	Seg. for HDR-BT
Xu *et al*.^19^	1	CTA	No	Yes	Visceral vsl.	Endovascular planning
Radl *et al*.^11,20^	56	CTA	No	Yes	Aorta + branches	Aortic tree seg.
Koitka *et al*.^21,22^	900	CT	Mixed	No	—	Body region seg.
Akilandeswari *et al*.^4^	825	CT	Yes	No	—	CRC polyp seg.
Ma *et al*.^12^	32	CT	No	Yes	Visceral branches	Vessel seg. + topo.
Sahoo *et al*.^9^	232	CT	Yes	No	—	CRC detect. (YOLOv8)
Zhang *et al*.^13^	11	CTA	No	Yes	SMA + branches	SB vascular guide
Flor *et al*.^14^	n/a	CE-CTC	Yes	Yes	Mesenteric vsl.	3D vessel-bowel map
Yao *et al*.^3^	1196+test	CECT	Yes	No	—	CRC detect. DL method (no bowel prep)
Raju *et al*.^23^	n/a (image-based)	Colonoscopy	Yes	No	—	Polyp/tumor cls.
La Barbera *et al*.^24^	79	CECT (ped.)	No	Yes	Ureters, aa., vv.	Pediatric ceCT seg.
Rudie *et al*.^25,26^	4274	CT	No	No	—	Organ-level ann. (trauma), (RSNA RATIC)

*Abbreviations:* seg. = segmentation; vv. = veins; vsl. = vessels; Abd. = abdominal; Reg. = registration; topo. = topology; detect. = detection; SB = small bowel; cls. = classification; aa. = arteries; FLR = future liver remnant; HDR-BT = high-dose-rate brachytherapy; CE-CTC = contrast-enhanced CT colonography; CECT = contrast-enhanced CT; ped. = pediatric; ann. = annotations.

Publicly available datasets focusing on abdominal vascular anatomy and CRC vary markedly in scope and clinical applicability. Most collections emphasize either oncologic characterization without detailed vascular mapping, or vascular segmentation unrelated to colorectal surgery.

Early work by Schenk *et al*.^8^ compared MRI and CT imaging for liver transplantation planning, providing paired datasets of portal and hepatic veins. While methodologically significant for validating cross-modal vessel analysis, it is unrelated to CRC. Conversely, Tong and Li^1^ presented a large cohort of stage II CRC patients with preoperative CT, but without vascular annotations, limiting its utility for surgical vascular planning. Similar oncologic datasets, such as those by Akilandeswari *et al*.^4^, Sahoo *et al*.^9^, and Yao *et al*.^3^, focus primarily on tumor or polyp detection using deep learning models, without providing arterial or venous segmentation.

Datasets integrating vascular analysis are more limited and often target hepatic or aortic systems. Simpson *et al*.^2^ offer portal-phase CT data with hepatic and portal vein segmentations in CRC liver metastases, supporting radiomics and prognostic modeling rather than resection planning. Criscuolo *et al*.^10^ and Radl *et al*.^11^ contribute vascular landmarks of aortic branch segmentations for algorithm validation and segmentation benchmarking, but without colorectal focus. Ma *et al*.^12^ and Zhang *et al*.^13^ provide detailed visceral arterial reconstructions (e.g., celiac trunk, SMA), useful for mesenteric studies yet not specific to CRC surgical anatomy. Flor *et al*.^14^ is notable for combining vascular maps with CT colonography to aid laparoscopic colorectal surgery but remains limited in scale and generalizability.

Few datasets link detailed vascular anatomy to oncologic context Yang *et al*.^15^ segmented portal and hepatic veins for navigation-guided brachytherapy of CRC liver metastases, yet the sample size was small. More recent resources such as SAROS [8] and RATIC [17] offer large-scale abdominal CT data but lack fine vascular detail required for vessel-aware colorectal planning.

Collectively, existing resources either lack vascular annotation or focus on extra-colonic territories. The present dataset provides comprehensive dual-phase CT segmentations of colonic arterial and venous anatomy validated by colorectal surgeons. It is intended to support the development and evaluation of automated methods for vascular recognition in preoperative CRC resection planning and to complement existing datasets relevant to abdominal vascular imaging.

## Methods

### Ethics statement

The study was approved by the Ethics Committee of the Department of Surgery, University Hospital Ostrava (approval number 117/2025). The committee granted permission for the retrospective use of routinely acquired clinical imaging data and issued a waiver of individual informed consent for research purposes. We confirm that all methods were carried out in accordance with relevant guidelines and regulations.

Participants were not actively recruited for this study. The dataset was retrospectively compiled from consecutive adult patients who underwent routine contrast-enhanced abdominal CT examinations at University Hospital Ostrava over a one-year period as part of standard clinical care.

All patients provided standard clinical informed consent prior to undergoing CT examination, which is routinely required at the institution. This general consent covers the performance of the imaging procedure and allows the use of fully anonymised clinical data for institutional quality control and clinical research purposes. For this specific retrospective study, the Ethics Committee waived the requirement for obtaining additional individual informed consent for research use and data sharing, due to the exclusive use of anonymised imaging data.

No additional informed consent was obtained from individual participants for this study. Instead, the Ethics Committee approved the retrospective use of routinely acquired clinical imaging data and formally waived the requirement for individual informed consent, in accordance with national regulations and institutional policies governing secondary use of anonymised clinical data.

Comprehensive safeguards were implemented to protect participant privacy and confidentiality. All imaging data were fully anonymised prior to analysis and public release, including removal of direct identifiers (e.g., names, dates of birth, patient identifiers) and all potentially identifying DICOM metadata. Each case was assigned a study-specific, non-reversible code. Data access during curation, annotation, and processing was restricted to authorised personnel within secure institutional infrastructure. Only anonymised imaging volumes and derived segmentation masks are publicly shared; no clinical reports, free-text information, or identifiable demographic data are included.

### Dataset Compilation

We compiled a dataset of 60 adult patients with the aim of achieving diversity and including the most common diagnoses that may affect visceral blood vessels. To ensure a broad spectrum of radiological findings, we did not limit the selection to patients with colorectal carcinoma alone. Instead, we randomly selected 60 patients who underwent contrast-enhanced CT (CECT) of the abdomen at our institution over a one-year period.

Only patients with either (a) native and venous phase scans or (b) native, arterial, and venous phase scans were included. Examinations performed with a polytrauma CT protocol were excluded, as this protocol merges arterial and venous perfusion into a single phase, often insufficiently, and these scans are never used for preoperative planning. Among the included patients, 10 had only native and venous phase scans, while 50 had both arterial and venous phases. When chest CT scans were also available, only the region of interest (ROI) from the diaphragm to the pelvis was used.

All scans were acquired using a Siemens Somatom Force scanner (2 × 192 detector rows, dual-energy capable). The slice thickness was typically 0.75 mm. Arterial phases were obtained 25–30 seconds after contrast injection, and venous phases 55–60 seconds after injection. The contrast agents used were either Visipaque 320 or Omnipaque 325, administered intravenously through a peripheral vein in the forearm or cubital region. Each CT contained approximately 700–900 axial slices.

The patient cohort consisted predominantly of Caucasians, with 13 Roma patients included. Ages ranged from 32 to 89 years. There were 37 males (mean age 61 years, mean BMI 34.4) and 23 females (mean age 63 years, mean BMI 32.6).

The dataset is designed for clinical application and, as such, represents patients who require medical intervention rather than constituting an anatomical study of normal human physiology. Consequently, patients with co-morbid conditions—including prevalent forms of cancer—were intentionally included. A subset of these patients had previously undergone cancer-related surgical procedures. This inclusion was deliberate, reflecting the progressive increase in patient age and the corresponding rise in the prevalence of comorbidities in clinical populations. Although prior treatments may result in alterations to vascular anatomy, recognition of these variations is of significant clinical relevance. Accordingly, training a neural network exclusively on data from healthy individuals would offer limited utility in real-world clinical practice.

### Clinical characteristics

- 20 patients had active oncological disease at the time of CT (including hepatic tumors, lung metastases, ovarian cancer, and lymphadenopathy from leukemia, breast cancer metastases, pancreatic cancer, bladder carcinoma, renal carcinoma, ileocecal tumor, retroperitoneal tumor, and prostate cancer).
- 11 patients had previously undergone cancer resection (colon, rectum, testis, cecum, small bowel, kidney).
- Other findings included: one aortic aneurysm, 22 cases of fatty liver, three cases of ileus, five of diverticulosis, one of acute diverticulitis, two patients with physiologic CT exams, multiple liver and kidney cysts, three ventral hernias, five symptomatic inguinal hernias, five adrenal adenomas, three hydronephroses, one gallbladder hydrops, one horseshoe kidney, one nephroptosis, two pancreatic pseudocysts, two cases of ascites, two with dilated pancreatobiliary ducts, one abdominal wall abscess, one paracolic abscess, one case with both small and large bowel contrast filling, and one colostomy.
- Foreign material was present in several cases, including osteosynthetic material after lumbar stabilization (1 patient), drainage after pseudocyst (2 patients), and one knife blade following abdominal stab injury (1 patient).
- Splenomegaly was noted in two patients, and one patient showed signs of gastric ulcer.

### Quantitative Analyses of Arteries dataset

The quantitative analyses of arteries dataset was done on 50 patients. The dataset have average image dimensions of 512 × 510 × 757 voxels (median: 512 × 512 × 703 voxels), ranging from 508 × 408 × 563 to 512 × 512 × 1196 voxels. Cropping to the region of interest (ROI) adjusts the mean dimensions to 512 × 429 × 757 voxels (median: 512 × 425 × 703 voxels), with a range of 508 × 383 × 563 to 512 × 512 × 1196 voxels, primarily reducing variability in the y-axis, corresponding approximately to the abdominal region from the diaphragm to the pelvis. Voxel spacing is uniform in the z-direction (slice thickness) at 0.70 mm, while in-plane (x–y) spacing averages 0.82 ± 0.09 mm (median: 0.82 mm), ranging from 0.64 mm to 0.98 mm, resulting in physical scan sizes averaging 418 × 417 × 530 mm (median: 422 × 422 × 492 mm) and ranging from 330 × 286 × 394 mm to 500 × 500 × 837 mm. Intensity values, reported in Hounsfield units (HU), span  −1092 HU to 3449 HU (mean:  −454 HU  ± 495, median:  −347 HU), with foreground regions (non-zero labeled voxels) ranging from  −1011 HU to 1933 HU (mean: 219 HU  ± 113, median: 219 HU), reflecting enhanced vascular contrast whereas background regions exhibited mean values near  −542 HU  ± 496. The bit depth is inferred as 16-bit signed integer, accommodating the full HU range. The foreground label occupying a sparse 0.038% on average (median:0.034, range: 0.016%–0.076%).

### Quantitative Analyses of Veins dataset

The quantitative analyses of veins dataset was done on 50 patients. The dataset comprises CT scans with a median image shape of 512 × 512 × 698 voxels, with dimensions ranging from a minimum of 443 × 357 × 563 to a maximum of 512 × 512 × 1015 voxels, as determined by the data analysis. The voxel spacing varies, with a median of 0.829 mm  × 0.829 mm  × 0.7 mm (mean: 0.821 mm  ± 0.095 mm), ranging from 0.641 mm  × 0.641 mm  × 0.7 mm to 0.976 mm  × 0.976 mm  × 0.7 mm, resulting in physical sizes of approximately 424.5 mm  × 424.5 mm  × 488.6 mm (median). All images are single-channel (grayscale) with a bit depth inferred from the intensity range of  −1084 to 3071 Hounsfield Units (HU) with a mean of  −447 HU and standard deviation of  ≈495 HU, typical for 16-bit medical imaging data. The foreground intensity, representing the contrast-enhanced regions, ranges from  −1024 HU to 2779 HU, with a mean of 145.77 HU and a standard deviation of 56.23 HU (median 152.16 HU). The dataset includes binary labels (background and foreground), with the foreground occupying a median of 0.108% of the image volume, indicating sparse regions of interest, such as visceral blood vessels, within the scans.

### Annotation process

**Fig. 1:**
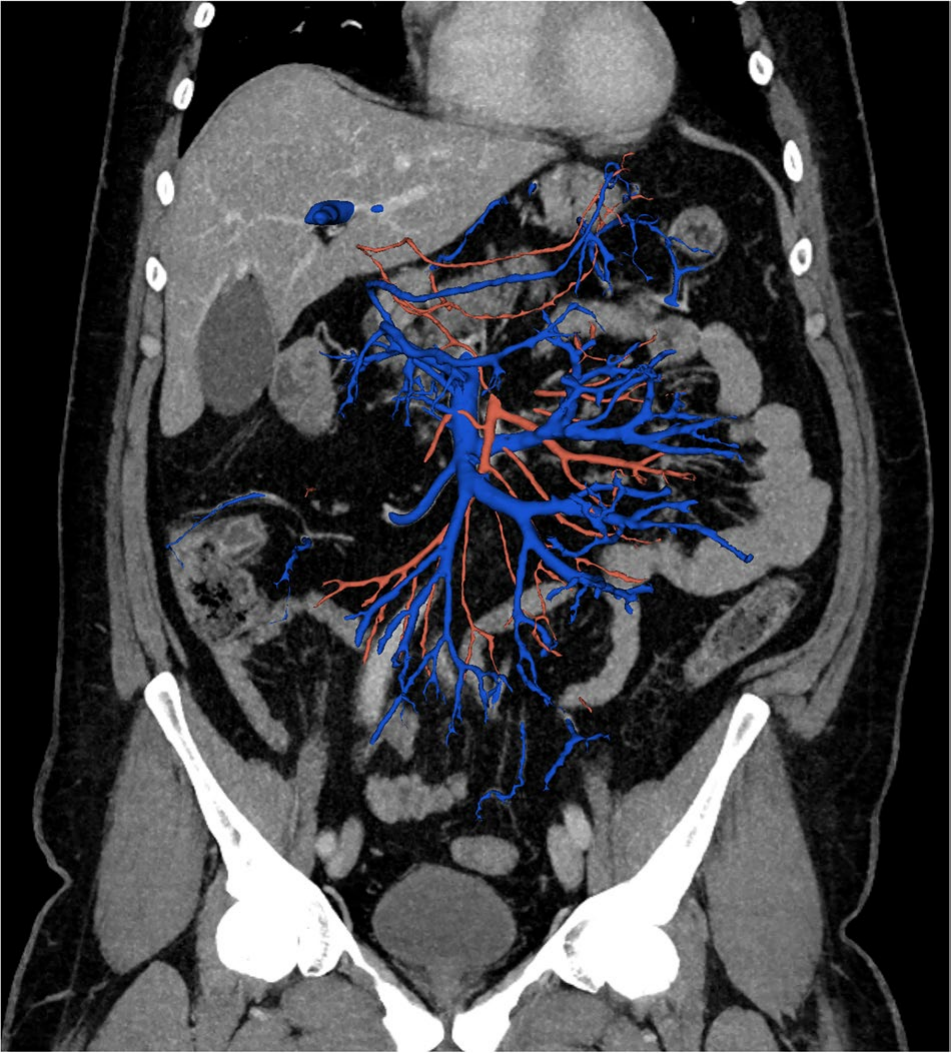
Manual mesenteric vessel annotations on dual-phase CT: arteries (red) from arterial phase and veins (blue) from venous phase, without inter-phase registration. Coronal and sagittal views highlight jejunal supply.

**Fig. 2:**
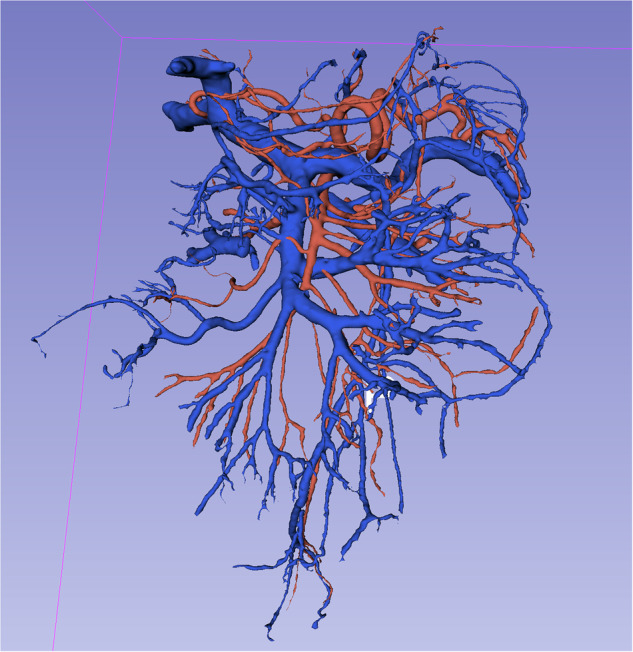
Dual-phase artery (red) and vein (blue) clarify mesenteric territories.

All segmentations were performed manually in 3D Slicer by an experienced surgeon and verified by a senior colorectal surgeon. Arterial phases were used to identify arterial structures, while venous phases were used to delineate veins. To minimize noise and preserve the original imaging characteristics, no registration between volumes was performed. The average segmentation time was 3 hours per CT phase. However, in some cases, the layout of both the phases was very similar and allowed us to grasp both arterial and venous compound of visceral vasculature within the same CT, although only for visual use, not for deep machine learning Fig. 1. The figure shows mesenterial arteries (red) and veins (blue) over the coronal CT reconstructions, focusing mainly on the blood supply of the jejunum. It is easier to comprehend than in pure 3D visualization—Fig. 2.

### Annotated vascular structures: Arteries

- Celiac trunk and branches: inferior phrenic, splenic, left gastric, common hepatic (including gastroduodenal, superior pancreaticoduodenal, right gastro-omental, and proper hepatic arteries with right and left branches). The right gastric artery was rarely visible. Occasionally, short gastric, left gastro-omental, and omental branches were identified.
- Superior mesenteric artery (SMA) and branches: jejunal and ileal arteries (commonly), ileocolic artery (nearly constant), right colic artery, right supracolic artery, inferior pancreaticoduodenal arteries, middle colic artery and its branches. Given the variability in nomenclature for right colon blood supply, we applied simplified terms: “right colic artery” for any major branch heading from the superior mesenteric artery or the ileocolic artery to ascending colon branch and “right supracolic artery” for a major branch supplying the hepatic flexure.
- Inferior mesenteric artery (IMA) and branches: left colic (often duplicated), sigmoidal arteries, and superior rectal artery.
- Other arteries: renal and, where identifiable, gonadal arteries.

### Veins

- Splenic vein and tributaries: left gastric vein (constant), right gastric vein and left gastro-omental vein (rare), short gastric veins (frequent).
- Inferior mesenteric vein (IMV): left colic, sigmoid, and superior rectal veins.
- Superior mesenteric vein (SMV) and tributaries: duodenal, jejunal, ileal, superior pancreaticoduodenal veins, Henle’s trunk (identified in most patients) with right gastro-omental, inferior pancreaticoduodenal, and right supracolic veins. Ileocolic, right colic, and middle colic veins were consistently identified. As with arteries, simplified terminology analogous with the arteries was applied for right colon venous drainage as well.
- Renal veins: consistently visible, with frequent left gonadal and left inferior suprarenal veins. Rare findings included retrocaval left renal vein and right gonadal vein.

**Fig. 3:**
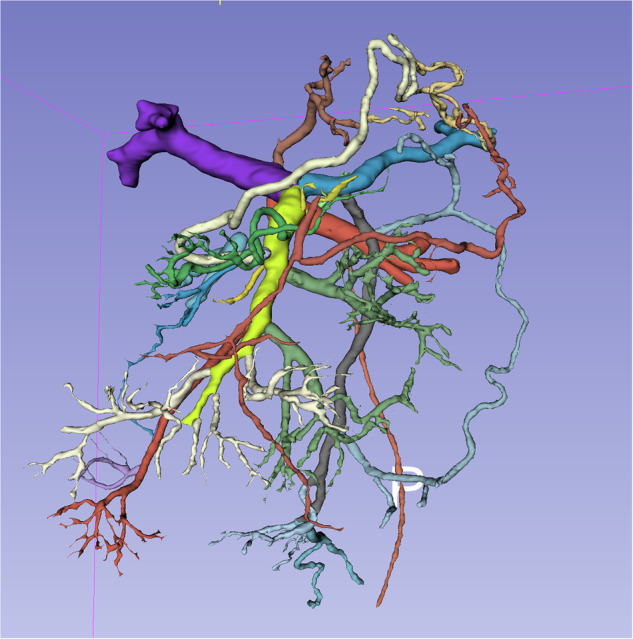
3D vascular mask with separate vessel labels. Venous tributaries to the inferior mesenteric (grey), superior mesenteric (yellow), and splenic (blue) veins are shown uniting into the portal vein (dark purple).

**Fig. 4:**
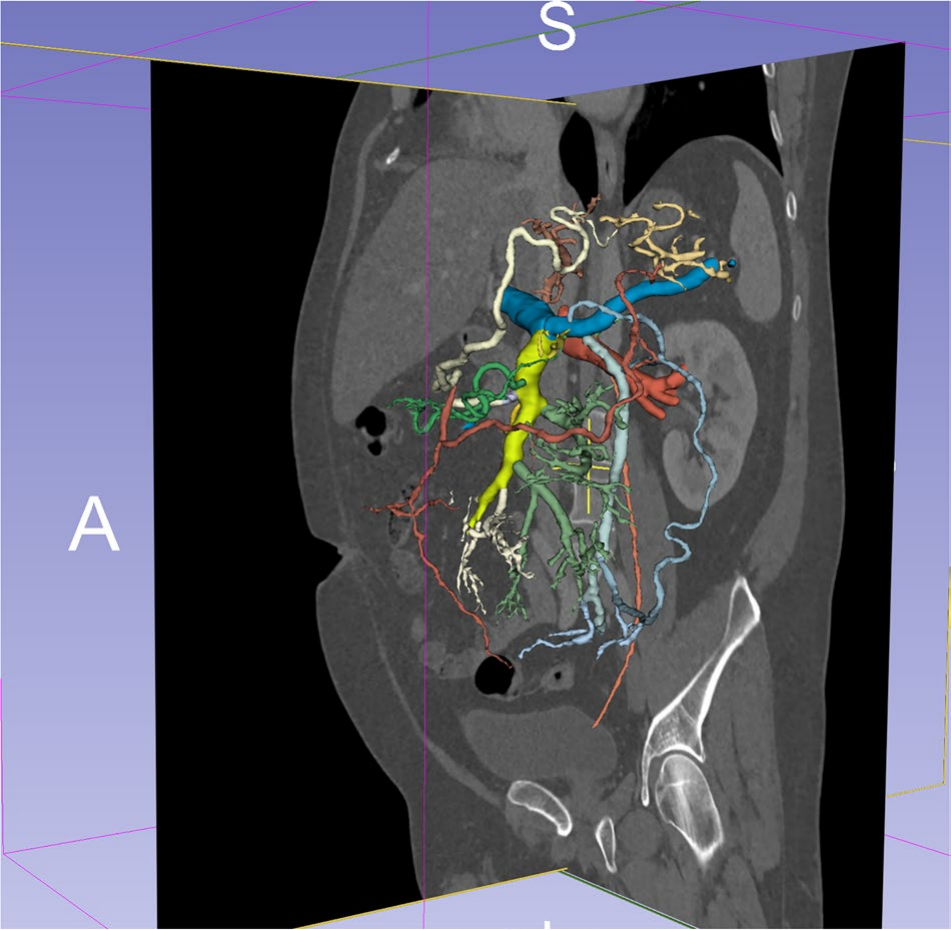
Sagittal and coronal CT overlays of the venous map (colors as in Fig. 3) for clearer spatial context; large vessels may be semi-automated, whereas segments lesser than 4 mm typically require manual delineation.

In the 3D mask, we created separate labels for each of the vessels above, and then a segment of all the venous and arterial vessels to depict them as a whole. One picture speaks a thousand words and the whole 3D mask is clearly depicted in the Fig. 3. The mask comprises individual veins, each with a different color, as tributaries to the inferior mesenteric vein (grey), superior mesenteric vein (yellow) and splenic vein (blue), that unite and finally form the portal vein (dark purple). The same veins can be seen overlying the sagittal and coronal CT reconstruction for a better understanding—Fig. 4 Generally speaking, the large vessels with a good contrast filling could be sometimes segmented using automated 3D Slicer tools. However virtually anything smaller than 4 mm in diameter had to be highlighted manually due to insufficient contrast difference between the surrounding tissue and their lumen.

Aiming at everyday practice in oncosurgery, not all vessels bear the same importance. The ground truth reflects clinically meaningful vascular anatomy rather than exhaustive segmentation of all distal micro-vascular branches. Special care was dedicated to the arteries and veins of the colon, pancreas, spleen, kidneys, stomach and liver. Blood vessels for the small intestine do not pose the same clinical significance because of their redundancy and a limited amount of surgery performed on the small intestine. Therefore, their segmentation was not as extensive as for other organs. We still made sure to depict every major ileal and jejunal branches of SMA and tributaries of SMV, although not always necessarily within their full course and ramification.

### Summary

Despite being limited to 60 patients, this dataset encompasses a wide variety of common abdominal pathologies and vascular patterns. Masks of venous structures were annotated in all 60 venous-phase scans, and arterial masks in 50 arterial-phase scans. The resulting 3D models were used to train a deep learning model for automated visualization of the abdominal arterial and venous networks as a whole, not restricted to the colorectum.

**Fig. 5:**
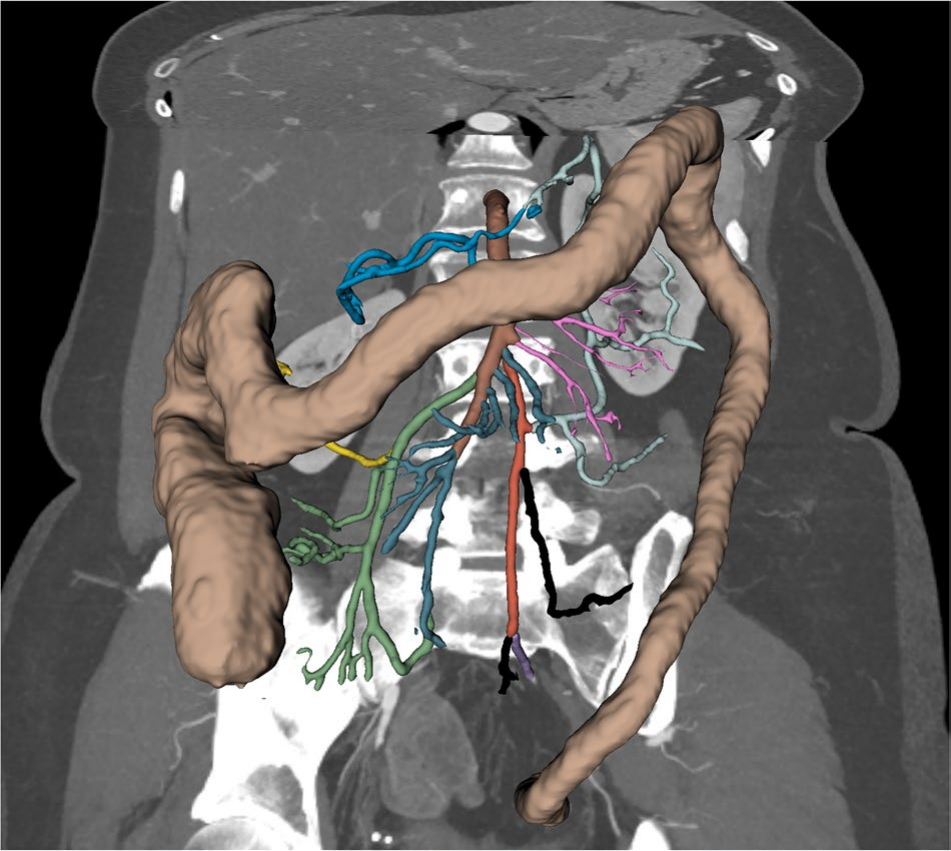
Patient-specific colonic arterial map: SMA (brown) with ileocolic (green), right colic (yellow), middle colic (royal blue), ileal (blue), and jejunal (pink) branches; IMA (red) with superior rectal (violet) and sigmoid (black) branches; left colic (light blue) anastomosis with middle colic (Riolan’s arcade).

An example of a sought after application is a Fig. 5: patient-specific vascular model depicting major arteries and their branches supplying the colon: superior mesenteric artery (brown), ileocolic artery (green), right colic artery (yellow), middle colic artery (royal blue), branches for ileum (blue) and jejunum (pink), inferior mesenteric artery (red), superior rectal artery (violet), branches for sigmoid colon (black), and left colic artery (light blue) anastomosing with the royal blue middle colic artery (Riolan’s arcade or Haller’s anastomosis).

The clinical contribution of this tool lies in improved preoperative planning. Familiarity with individual vascular variations before surgery can enhance patient safety and oncological outcomes, potentially reducing unexpected intraoperative bleeding and improving surgical radicality.

## Data Record

The dataset is available at 10.5281/zenodo.17407158. The repository^16^, titled ColonVessels 2026: A Colon Vessels Arteries and Veins Segmentation Dataset, contains 60 CECT studies: 50 dual-phase and 10 venous-only. Dual-phase studies include arterial and venous CT volumes in .nrrd format, along with corresponding arterial and venous segmentation masks in .seg.nrrd format. Venous-only studies provide venous-phase CT volumes and vein segmentation masks.

All data are organized into patient-specific folders following the pattern pat_XXX/. Each folder contains the imaging volumes and associated labels for that patient. Examples include:

- **Dual-phase cases (pat_001–pat_050):**
  - pat_XXX_Arterial_Phase_CT.nrrd
  - pat_XXX_Arterial_Phase_Arteries.seg.nrrd
  - pat_XXX_Venous_Phase_CT.nrrd
  - pat_XXX_Venous_Phase_Veins.seg.nrrd
- **Venous-only cases (pat_051–pat_060):**
  - pat_XXX_Venous_Phase_CT.nrrd
  - pat_XXX_Venous_Phase_Veins.seg.nrrd

A README file in the repository provides an overview of the directory structure, file naming conventions, and guidance on loading the data. All imaging and segmentation files have been fully anonymized in accordance with institutional and ethical data protection requirements.

Patients pat_001 to pat_050 were used for technical validation. Of these, pat_001–pat_045 served as the training set, and a subset of these cases was used for validation during model development. Patients pat_046–pat_050 were reserved for testing.

## Technical Validation

**Fig. 6:**
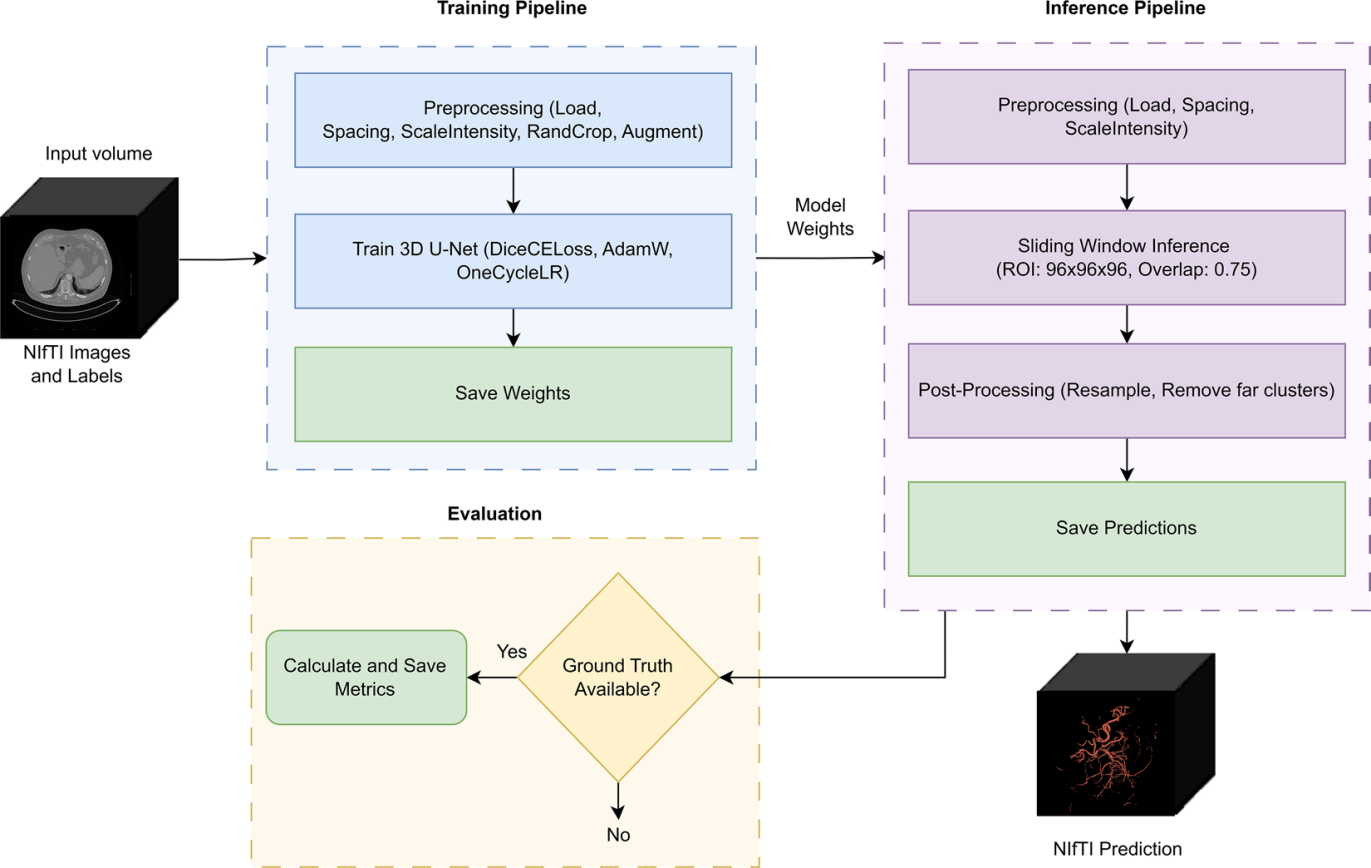
Workflow for colon vessel segmentation.

The following sections present a detailed analysis of a colon vessel (arteries and veins) segmentation pipeline developed using the MONAI framework^17^ with a U-Net architecture^7^. The methodology in Fig. 6 includes a comprehensive description of the algorithm, including data preprocessing, model architecture, and training procedure. We provide 2D and 3D visualizations comparing manual annotations by physicians, algorithm outputs, and their fusion to demonstrate agreement. Quantitative evaluation using metrics is included to assess segmentation performance across various test cases.

### Data Split

The dataset^16^ comprises CT volumes from a total of 50 patients. Out of these, 45 patient scans are used for training. During model development, 20% of the training set (i.e., 9 patients) are randomly selected for validation to support hyperparameter tuning and prevent overfitting. The remaining 5 patient scans are reserved exclusively for testing and are used to provide an unbiased evaluation of the trained model’s performance.

### Data Preprocessing

As a first step, all .nrrd images and segmentation masks were converted to NIfTI format. All images and corresponding masks were then preprocessed using the MONAI transformation framework to ensure spatial consistency, intensity normalization, and enhanced model generalization through data augmentation.

For **training**, the preprocessing and augmentation pipeline consisted of the following steps to improve model generalization and mitigate overfitting:

- LoadImaged: Loads the CT volumes and corresponding annotations in NIfTI format.
- EnsureChannelFirstd: Converts each image and label into a channel-first format (*C* × *D* × *H* × *W*) for compatibility with the U-Net model architecture.
- Spacingd: Resamples both images and labels to an isotropic voxel spacing of 0.7 mm. Bilinear interpolation is applied to the image data, and nearest-neighbor interpolation is used for segmentation masks to preserve discrete label values.
- ScaleIntensityRanged: Normalizes CT intensities from the Hounsfield unit range [−1000, 1000] to a standardized [0, 1] range to ensure consistent model input.
- RandCropByPosNegLabeld: Randomly extracts 140 patches of size 96 × 96 × 96 voxels per volume, maintaining a balance between vessel (positive) and non-vessel (negative) regions.
- RandRotate90d: Applies random 90^∘^ rotations along the axial plane with a probability of 0.5 to increase rotational robustness.
- RandFlipd: Randomly flips the input along the axial axis with a probability of 0.5 to enhance spatial invariance.
- ToTensord: Converts the processed data into PyTorch tensors for efficient loading and GPU computation.

For **validation**, the same preprocessing pipeline was used, except that random augmentations (RandRotate90d and RandFlipd) were disabled to ensure deterministic evaluation. A fixed number of 10 patches per volume were extracted uniformly (RandCropByPosNegLabeld) to maintain consistency across validation samples.

For **testing**, the preprocessing followed the same deterministic configuration as validation, with the exception that RandCropByPosNegLabeld was omitted.

### Model Architecture

The U-Net model^7^ is a 3D convolutional neural network with the following configuration:

- Input Channels: 1 (grayscale CT volume).
- Output Channels: 2 (background and vessel classes).
- Channels: (16, 32, 64, 128, 256) across five encoder-decoder levels.
- Strides: (2, 2, 2, 2) for downsampling/upsampling.
- Residual Units: 2 per level for improved gradient flow.

**Fig. 7:**
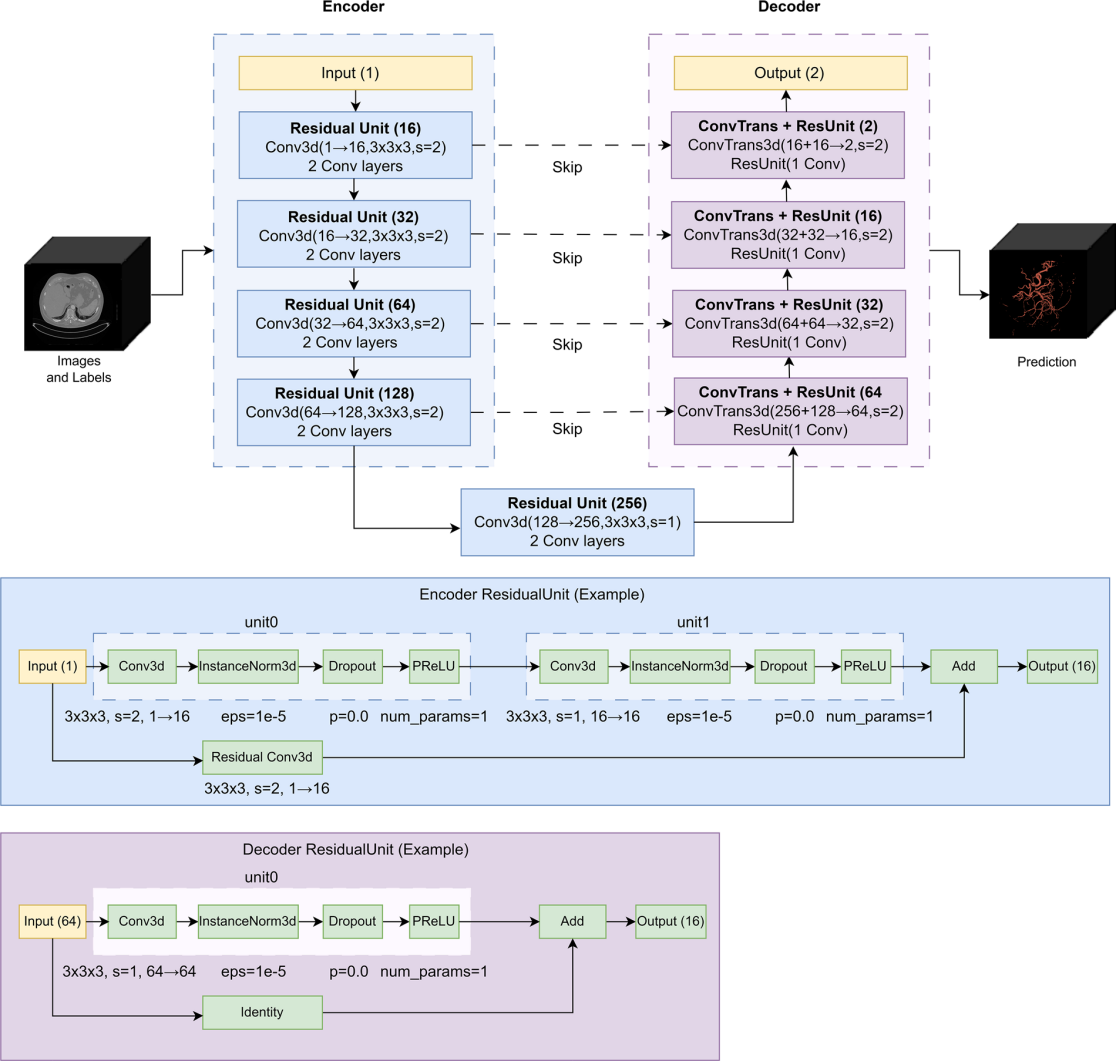
Architecture of the MONAI 3D UNet.

The MONAI U-Net implementation (Fig. 7) is a fully 3D encoder–decoder architecture tailored for high-resolution volumetric segmentation. It processes single-channel CT volumes to generate binary vessel segmentation maps through a symmetric five-level hierarchy, where each encoder stage begins with downsampling via strided 3 × 3 × 3 convolutions (stride = 2), progressively reducing spatial dimensions by a factor of 16 while doubling feature channels. Within each level, two residual units—each composed of sequential 3 × 3 × 3 convolutions with instance normalization and PReLU activation followed by identity addition—enhance feature refinement and gradient propagation. The bottleneck layer applies a similar residual block (stride = 1) to capture rich semantic context at the coarsest scale. In the decoder, transposed convolutions (stride = 2) upsample feature maps, which are then concatenated with corresponding high-resolution encoder features via MONAI’s SkipConnection, enabling precise localization by fusing contextual and spatial information. Each decoder stage mirrors the encoder with two residual units, halving channel counts per level. The architecture is recursively constructed: each block assembles a downsampling path, recursively builds the deeper sub-network, and attaches an upsampling path with skip integration. A final 1 × 1 × 1 convolution maps the restored 16-channel feature volume to the two-class output, yielding full-resolution probabilistic segmentations. This design—leveraging early strided sampling, residual learning, and structured skip connections—ensures numerical stability, memory efficiency, and superior boundary delineation.

### Training Procedure

#### Loss function

The loss function is a combination of Dice Loss and Cross-Entropy Loss (DiceCELoss), applied after softmax activation and one-hot encoding of labels. For a predicted segmentation $\widehat{Y}\in {{\mathbb{R}}}^{C\times D\times H\times W}$ and ground truth $Y\in {{\mathbb{R}}}^{C\times D\times H\times W}$, where *C* is the number of classes (*C* = 2 for background and vessel), and *ϵ* = 1 × 10^−5^ prevents division by zero, the loss functions are defined as follows:

$${\rm{DiceLoss}}=1-\frac{1}{C}\,\mathop{\sum }\limits_{c=1}^{C}\frac{2\sum _{d,h,w}{\widehat{Y}}_{c,d,h,w}\,{Y}_{c,d,h,w}+\epsilon }{\sum _{d,h,w}{\widehat{Y}}_{c,d,h,w}+\sum _{d,h,w}{Y}_{c,d,h,w}+\epsilon }\,.$$

The cross-entropy loss is

$${\rm{CELoss}}=-\frac{1}{D\,H\,W}\sum _{d,h,w}\,\mathop{\sum }\limits_{c=1}^{C}{Y}_{c,d,h,w}\,\log \,\left({\widehat{Y}}_{c,d,h,w}\right).$$

With weights *λ*_dice_ = 1.0 and *λ*_ce_ = 1.0, the combined loss is

$${\rm{Loss}}={\lambda }_{{\rm{dice}}}\cdot {\rm{DiceLoss}}+{\lambda }_{{\rm{ce}}}\cdot {\rm{CELoss}}.$$

#### Optimization and Training Settings

**Fig. 8:**
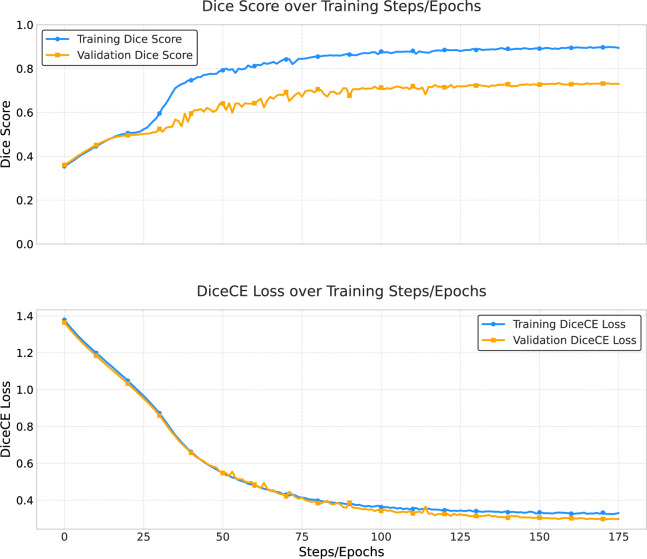
Training and validation Dice score and DiceCE loss over epochs, showing model convergence and generalization performance.

The model is trained using the AdamW optimizer with an initial learning rate of 5 × 10^−4^ and a weight decay of 1 × 10^−4^ for regularization. A OneCycleLR scheduler adjusts the learning rate over 200 epochs, with a maximum learning rate of 5 × 10^−4^ and a 40% warm-up period. Training is performed on a GPU with a batch size of 2 and mixed precision to reduce memory usage. Early stopping is applied with a patience of 10 epochs based on validation loss. The patch size is 96 × 96 × 96. The Dice score and DiceCE loss is depicted in Fig. 8.

### Inference Procedure

The inference pipeline processes NIfTI-format CT images using the trained 3D U-Net model. The preprocessing pipeline prepares input images for inference using MONAI transforms, as explained in the data preprocessing section.

The model is loaded with pre-trained weights and set to evaluation mode. Inference is performed using a sliding window approach to handle large volumes. The key parameters are ROI Size 96 × 96 × 96 voxels, Batch Size 4 patches per sliding window iteration, Overlap 0.75 to ensure smooth transitions between patches. For a volume $I\in {{\mathbb{R}}}^{D\times H\times W}$, the model predicts a probability map $\widehat{P}\in {{\mathbb{R}}}^{C\times D\times H\times W}$, where *C* = 2. The final segmentation $\hat{Y}$ is obtained by applying argmax:

$$\hat{Y}=\mathop{{\rm{\arg }}\,{\rm{\max }}}\limits_{c}({\hat{P}}_{c}).$$

### Post-processing

Post-processing helps to improve predictions.

- **Resampling:** Predictions are ^ resampled to the original volume shape using nearest-neighbor interpolation via scipy.ndimage.zoom to align with the original image space and format.
- **Discretization:** The AsDiscreted transform converts probabilities to binary labels and one-hot encodes ground truth for metric computation.

### Evaluation

#### Segmentation Output Visualizations

**Fig. 9:**
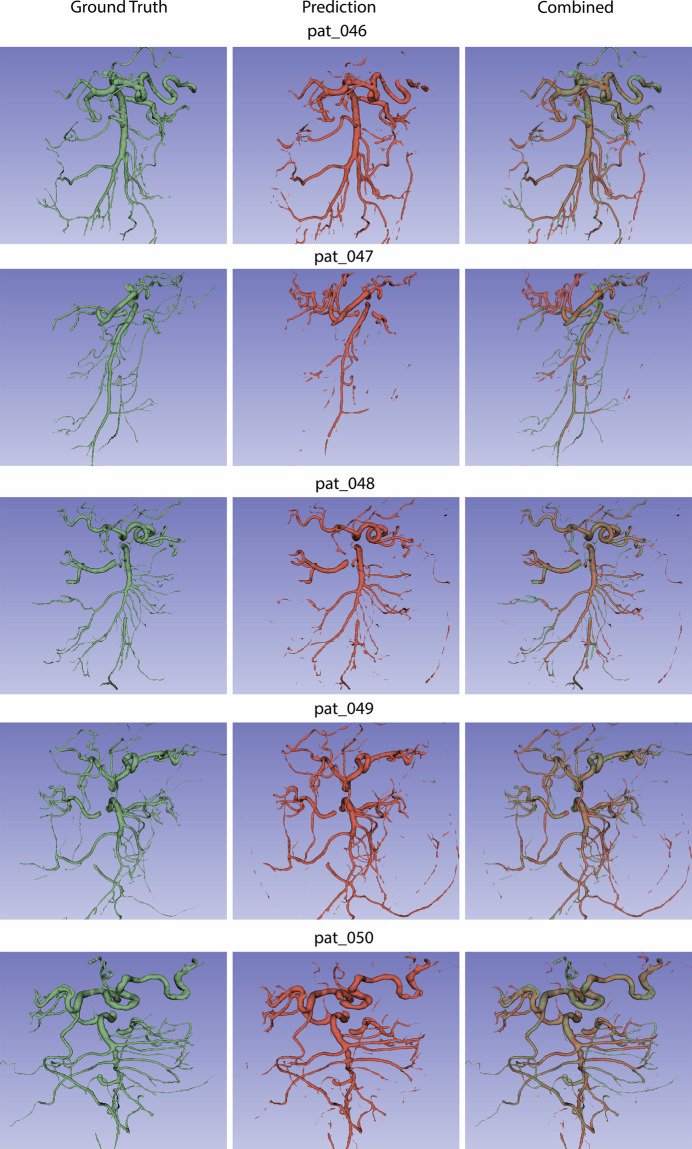
Arteries Segmentation Output. Overlay of the Manual annotation by a physician and the output of the algorithm to show the degree of agreement. Where: Ground truth (Green), Prediction (Red).

**Fig. 10:**
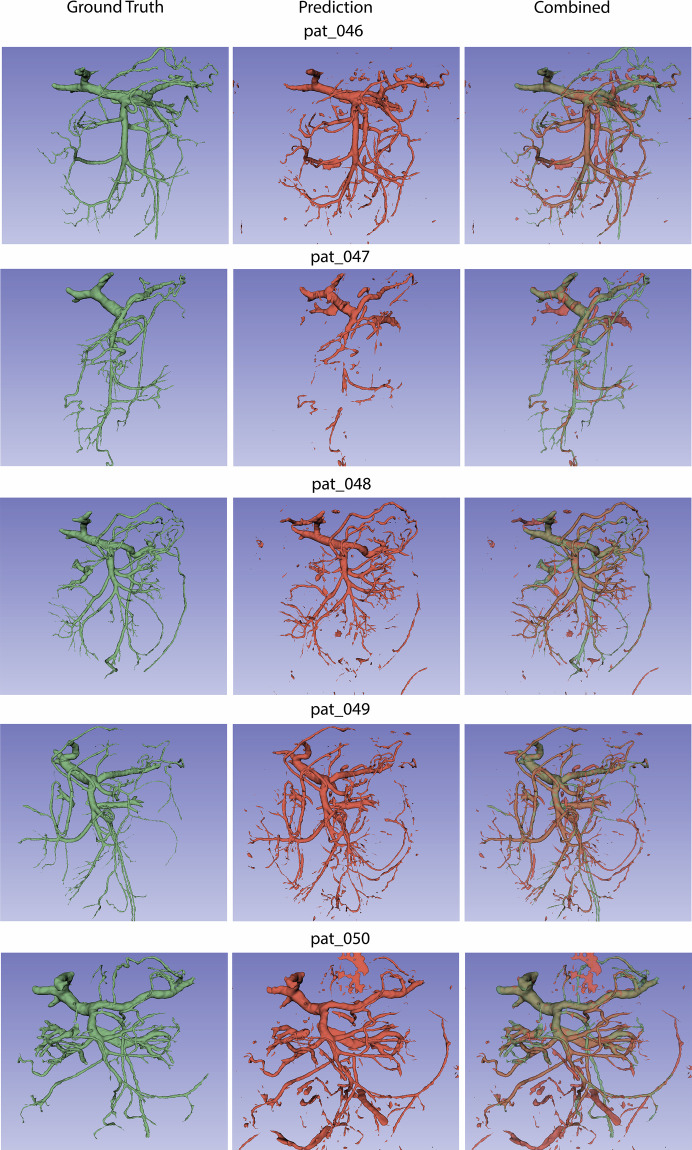
Veins Segmentation Output. Overlay of the Manual annotation by a physician and the output of the algorithm to show the degree of agreement. Where: Ground truth (Green), Prediction (Red).

To illustrate the algorithm’s performance, we provide 2D and 3D visualizations of segmentation results across multiple test cases. These visualizations Figs. 9, 10 demonstrate the algorithm’s ability to capture fine vascular details, with the overlay images indicating high agreement in most regions, though some discrepancies occur in areas with low contrast and very thin vascular structures.

#### Segmentation Output Quantitative Evaluation

Segmentation performance is evaluated using the Dice Similarity Coefficient (DSC) on a test set.

- **Dice Score:** A measure of overlap between predicted $(\widehat{Y})$ and ground-truth masks (*Y*):
  $${\rm{DSC}}=\frac{2\,| \widehat{Y}\cap Y| }{| \widehat{Y}| +| Y| }.$$
- **Sensitivity/Recall:** Measures how well the model identifies positive pixels:
  $${\rm{Sensitivity}}=\frac{TP}{TP+FN}.$$
- **Precision:** Measures how many predicted positive pixels are actually correct:
  $${\rm{Precision}}=\frac{TP}{TP+FP}.$$

The average DSC of 71.56% for arteries and 65.5% for veins indicates good overlap with manual annotations, see Tables 2, 3.

**Table 2:** Performance Metrics for Artery Segmentation Across Test Cases.

Patient Name	Dice	Sensitivity	Precision
pat_046	75.47	76.46	74.5
pat_047	59.08	55.73	62.86
pat_048	69.92	76.19	64.61
pat_049	78.21	83.89	73.25
pat_050	75.13	79.67	71.09
**Average**	**71.56**	**74.39**	**69.26**

**Table 3:** Performance Metrics for Vein Segmentation Across Test Cases.

Patient Name	Dice	Sensitivity	Precision
pat_046	65.98	71.47	61.27
pat_047	57.76	54.86	61.00
pat_048	68.97	76.43	62.85
pat_049	70.29	80.12	62.60
pat_050	64.71	77.33	55.63
**Average**	**65.54**	**72.04**	**60.67**

## Usage Notes

This dataset enables research on mesenteric vessel segmentation, vascular anatomy modeling, and surgical planning support using contrast-enhanced abdominal CT. The dual-phase studies support phase-specific or multi-phase learning approaches.

Segmentation masks are provided as 3D label maps and can be loaded directly in 3D Slicer, or converted to NIfTI for use in deep learning frameworks such as MONAI. Spatial alignment between images and labels should be preserved during preprocessing, especially when resampling or cropping.

Because arterial and venous phases were not inter-phase registered, the dataset is suitable for studying contrast-dependent vessel appearance and for developing cross-phase registration methods. Small vessels (<4 mm) may be incompletely delineated, and class imbalance between large vessels and distal branches should be considered when designing training strategies.

Data originate from a single scanner and institution, which may limit generalization. Domain adaptation may be required for external data. The provided training/testing split can serve as a reference benchmark, but alternative validation schemes may also be applied.

## Data Availability

The dataset is available at 10.5281/zenodo.17407158 under a CC BY 4.0 license.

## Code availability

The code used to perform inference on the test dataset is available at 10.5281/zenodo.17407158 under a CC BY 4.0 license.
